# Abnormal quantitative sudomotor axon reflex test results in patients with tinnitus as a possible indicator of small fiber neuropathy

**DOI:** 10.3389/fneur.2024.1297371

**Published:** 2024-02-05

**Authors:** Hye Lim Lee, Hyun Ji Lyou, Jae-Jun Song, Chi Kyung Kim

**Affiliations:** ^1^Department of Neurology, Korea University College of Medicine, Seoul, Republic of Korea; ^2^Department of Otorhinolaryngology, Korea University College of Medicine, Seoul, Republic of Korea

**Keywords:** tinnitus, quantitative sudomotor axon reflex test, heart rate variability, Valsalva ratio, small-fiber neuropathy, Tinnitus handicap inventory, autonomic function tests

## Abstract

A few cases of small fiber neuropathy (SFN) and tinnitus (TN) associated with coronavirus disease 2019 have been reported. However, the relationship between SFN and TN has not been studied. This study investigated a possible relationship between SFN and patients with TN (PwTNs) using autonomic function tests (AFTs) including quantitative sudomotor axon reflex tests (QSART). We performed QSARTs and other AFTs such as the Sympathetic skin response (SSR), Valsalva ratio (VR), and heart rate variability (HRV). The QSART results, obtained at seven hospitals using same protocols, were compared between PwTNs and healthy controls. We confirmed the abnormalities in SSR, VR, and HRV in PwTNs, although those parasympathetic AFTs were not performed in healthy controls. Additionally, we checked Tinnitus handicap inventory (THI) scores for PwTNs and ~50% of PwTNs had low-grade disability, whereas 9.3% had high-grade disability. Data from 57 PwTNs and 122 healthy controls were analyzed. The sweat volumes of QSART in the older age group tended to be higher in the PwTNs than in age-matched healthy controls, and significant differences between the PwTN and control groups were observed in the feet in both sexes (*p* < 0.001) and in the arms in women (*p* = 0.013). In the younger age group, the sweat volumes in the feet of men were higher in PwTNs than in healthy controls (*p* = 0.017). No association was observed between THI and QSART scores. In this study, the sweat volumes in QSARTs were higher in PwTNs than in healthy controls. However, abnormal SSR, HRV, and VR results were not commonly observed in PwTNs. Although the results should be interpreted with caution because of limitations in study, PwTNs might also have SFN apart from dysautonomia. This is the first study to perform QSART with other parasympathetic AFTs in PwTNs. However, larger and more rigorously controlled studies will be needed to reveal the relationship between SFN and TN in the future.

## 1 Introduction

Tinnitus (TN) is a subjective perception of internally generated and indistinct noises or tones without any external stimuli. Approximately 15% of population in America, Europe, and Asia may suffer from TN (1). Although TN is a common medical condition, its causes remain unclear. There are many hypotheses concerning the pathophysiology of TN, from the central nervous system to the peripheral nerves (2). Recently, associations with the autonomic nervous system (ANS) have been suggested, such as abnormalities in heart rate variability (HRV) (3–5).

During the coronavirus disease 2019 (COVID-19) pandemic, cases of TN and small fiber neuropathy (SFN), a type of peripheral neuropathy (PN), associated with COVID-19 have been reported (6, 7). TN can occur as a result of chemotherapy; previous studies have suggested that TN may be caused by PN, especially in the auditory nerve (8–11). Certain drugs, such as platinum or taxane agents, are concomitantly toxic to the auditory and peripheral nerves (12). However, SFN is different from these types of PN, which mainly affects small-diameter fibers in peripheral nerves, such as myelinated A-delta and unmyelinated C fibers, and is commonly comorbid with dysfunction of the ANS (13). While skin biopsy to assess intraepidermal nerve fiber density is the gold standard for diagnosing SFN, quantitative sudomotor axonal reflex tests (QSARTs) may be used as a useful diagnostic tool to check the function of small-diameter peripheral nerves (14). In addition, the QSART is useful for evaluating sudomotor function, a specialized postganglionic sympathetic cholinergic function in the autonomic function test (AFT). On the other hand, parasympathetic domains may be evaluated with other tools such as HRV or Valsalva ratio (VR). Although abnormalities in parasympathetic domains using HRV in patients with TN (PwTNs) have been reported, the possibility of concomitant dysautonomia in PwTN, however, remains controversial (3, 15). Moreover, the function of the sympathetic domain has not yet been evaluated in PwTNs. Thus, it is beneficial to perform QSART with other parasympathetic AFT to check for abnormalities of small-diameter fibers and dysfunction in the ANS.

SFN is usually defined as a dysfunction of small-diameter nerve fibers, suggested by distal axonal loss or neuronal degeneration, which could cause autonomic dysfunction (16). Cochlear neuronal degeneration or the loss of inner and outer hair cells was also reported as a possible mechanism for TN. Neuronal degeneration or axonal cell loss might be related common mechanism of two diseases. From this perspective, the performance of QSART would help reveal a shared mechanism between two diseases, SFN and TN.

We aimed to investigate the presence of QSART abnormalities in PwTN, including Sympathetic skin response (SSR), HRV, and VR to understand the shared mechanism for TN and SFN such as dysautonomia or PN. The results in QSART and other AFTs were compared between PwTNs and healthy controls.

## 2 Materials and methods

### 2.1 Participants

We enrolled consecutive PwTNs who visited the outpatient clinic of the Department of Otorhinolaryngology between July 2020 and December 2020. We collected clinical information regarding dizziness, hearing ability, and medical history, as well as the duration of TN and its impact on daily life. Patients aged >19 years were included, whereas those with underlying diseases, such as cancer, diabetes mellitus (DM), chronic alcoholism, vasculitis, hereditary neurologic diseases, and recent systemic infectious diseases, such as COVID-19, were excluded from the study. A total of 57 patients were enrolled in the PwTN group. We excluded patients with systemic diseases that could affect the results of AFTs (*n* = 11) and those aged < 30 years (*n* = 3) because of an inadequate number of subgroups for statistical analysis. The data for the healthy control group were obtained from a previous study that published reference QSART values for a South Korean population (17). The participants included in the study did not have any neurological or systemic disorders and were not receiving regular medications. Participants with disorders that could affect the autonomic nervous system, such as DM, alcoholism, malnutrition, and anti-cancer therapy, were excluded. We selected 122 participants who were age-matched with PwTNs from 167 volunteers who underwent testing.

Patients and healthy controls were categorized into the following four groups based on age and sex: 30–49 years, men; 30–49 years, women; >50 years, men; and >50 years, women. The QSART and Tinnitus handicap inventory (THI) test were performed in all patients. PwTNs were surveyed about past medical and social histories, including the level of stress. The requirement for informed consent was waived, and the study was approved by the Institutional Review Board of Korea University Medicine (No. K2023-1477-002).

### 2.2 The QSART and other AFTs

In this study, QSART was performed on the medial forearm (three-quarters of the distance from the ulnar epicondyle to the pisiform bone), proximal lateral leg (5 cm distal to the fibular head), distal medial leg (5 cm proximal to the medial malleolus), and proximal foot (above the extensor digitorum brevis muscle). The test was performed using the Q-Sweat quantitative sweat measurement system (WR Medical Electronics Co., Maplewood, MN, USA). QSART tests were performed using the same methods and the Q-Sweat quantitative sweat measurement system in the PwTN and healthy control groups at seven hospitals at different times. We also performed the SSR test and other AFTs to calculate HRV and VR using an electromyographic system. The heart rate was measured during 5 s of inhalation and 5 s of exhalation, and these measurements were repeated eight times. After a 2-min break, an additional test was performed using a similar protocol. The five largest differences in heart rate during inhalation and exhalation from the first eight measurements were selected and averaged. HRV was calculated using HRV analysis software in a group of healthy controls and manually calculated in PwTNs. VR was determined by measuring the blood pressure and heart rate while blowing into a mouthpiece at a pressure of at least 40 mmHg for 15 s. VR was calculated by dividing the highest heart rate during phase 2 by the lowest heart rate during phase 4 in the healthy control group. In the PwTN group, VR was calculated from the heart rate ratio using electromyographic equipment without blood pressure monitoring; SSR was recorded using electromyography equipment in PwTNs. Electrodes were placed on the palms and feet, and electrophysiological responses to electrical stimuli were recorded (18).

We categorized patients into four subgroups based on age (younger group: 31–49 years; older group: ≥50 years) and sex in both groups of PwTNs and healthy controls. The QSART, HRV, VR, and SSR values in both groups were determined according to reference values. Based on the age- and sex-specific reference range of the Korean population, we considered the results in the lower 5 percentile or upper 5 percentile of the QSART as functional abnormalities (19). The length-dependent pattern (LDP) in the QSART was checked, which was defined as a distal sweat volume below one-third of the proximal value (20). In tests of HRV and VR, results in lower 5% was considered a functional abnormality. For interpretation of the SSR, those showing one or more sites without wave formation were considered abnormal. The results of QSART were compared between the healthy control and PwTN groups; however, the results of HRV and VR were not compared because of the differences in the methods used in both the groups, and SSR was performed in PwTN.

### 2.3 Tinnitus handicap inventory

The THI can be used to measure the impact of TN on daily life. It consists of a 25-item inventory divided into three subscales: functional (11 items), emotional (9 items), and catastrophic (5 items) (21). Every item is assigned 4 points; therefore, the total THI score ranges from 0 to 100 points. We used the Korean version of the THI, whose validity and reliability have been previously demonstrated (21). The total scores were used to grade patients with and without hearing loss (22) resulting in four groups: low (0–22 points in the normal hearing group and 0–24 points in the hearing loss group), lower moderate (22–46 points in the normal hearing group and 26–48 points in the hearing loss group), upper moderate (48–70 points in the normal hearing group and 50–74 points in the hearing loss group), and high (72–100 points in the normal hearing group and 76–100 points in the hearing loss group).

### 2.4 Audiometry

Only participants in PwTN performed audiometry at the time of diagnosis with TN. We checked the presence of sensorineural hearing loss (SNHL), and it was defined that air and bone conduction curves worsened-30 decibels or greater over at least three contiguous audiometric frequencies- without air-bone gap on audiogram of pure tone audiometry (PTA) (23). SNHL Based on the results of PTAs, the values of air conduction (AC) threshold were collected in both sides.

### 2.5 Statistical analysis

Comparisons between categorical and continuous variables were performed using the chi-square test or independent Student's *t*-test. Based on the normality test results, the Mann–Whitney *U*-test was used to compare age- and sex-matched PwTNs to healthy controls without correction for multiple comparisons. All statistical analyses were performed using SPSS (version 29.0, IBM Corp., Armonk, NY, USA). All significant results were set at a two-tailed *p* < 0.05.

## 3 Results

### 3.1 Clinical characteristics

A total of 165 patients were included in this study. Initially, 57 patients were enrolled in the PwTN group; however, we excluded patients 14 patients (systemic diseases, *n* = 11 and age < 30 years, *n* = 3). In the control group, 122 individuals aged > 30 years were selected from a previously published study (17). The mean age of PwTNs was higher than that of healthy controls (*p* < 0.001; Table 1). However, no difference in age was observed between the age-matched and sex-matched groups (men: 30–49 years, *p* = 0.214; men: >50 years, *p* = 0.185; women: 30–49 years, *p* = 0.117; women: >50 years, *p* = 0.061). Approximately 30% of PwTNs reported dizziness and 80% were documented sensorineural hearing loss in the pure audiometry test. Around 10% of PwTNs were surveyed with some or severe stress.

**Table 1:** Demographics in tinnitus and control group.

	**Tinnitus**	**Control**	***p*-value**
	**(*****N*** = **43)**	**(*****N*** = **122)**	
Age, Mean (SD)	57.5 (12.1)	48.7 (11.2)	< 0.001
Female, n (%)	24 (55.8)	86 (70.5)	0.889
**QSART**			
Arm	1.1 (0.9)	0.6 (0.7)	0.001
Proximal Leg	1.2 (0.7)	1.1 (0.8)	0.335
Distal Leg	1.0 (0.7)	0.9 (0.7)	0.208
Foot	0.9 (0.7)	0.3 (0.3)	< 0.001
**Tinnitus handicap index (*****N*** = **32)**			
**Scale score, mean (SD)**			
Functional domain	13.4 (11.8)		
Emotional domain	13.2 (10.3)		
Catastrophic domain	7.1 (6.1)		
Sum of score	33.6 (25.4)		
**Grade**, ***n*** **(%)**			
Low	15 (46.9)		
Lower moderate	10 (23.3)		
Upper moderate	4 (12.5)		
High	3 (9.3)		
**Clinical Characteristics (*****N*** = **43)**			
Dizziness, *n* (%)	12 (27.9)		
Sensorineural hearing loss, *n* (%)	34 (79.1)		
Stress, *n* (%)	5 (11.6)		

QSART, Quantitative sudomotor axon reflex test; SSR, Sympathetic skin response; HRV, Heart rate variability; VR, Valsalva ratio.

### 3.2 Tinnitus handicap inventory

For PwTNs, the mean total THI score was 33.6 (standard deviation: 25.4) points. The patients were categorized into four groups based on their total scores. Approximately half of PwTNs were classified as having a low grade of disability, whereas 9.3% of patients were classified as having high-grade disability. No association was observed between the total THI scores and QSART results, HRV, or VR in the correlation analysis (*p* = 0.117, *p* = 0.953, and *p* = 0.537, respectively). We analyzed the association between each domain of the THI and the QSART results, HRV, and VR; however, no association was noted among them in statistics.

### 3.3 QSART and other AFTs

The sweat volumes measured by QSART did not differ between PwTN and healthy controls at the arm, proximal leg, and distal leg sites. However, the sweat volumes obtained for the foot were higher in the TN group than in the healthy control group (Table 2). Based on reference values for Koreans, ~52% (*n* = 24) of PwTNs showed abnormal results in QSART (30–49 years: 6/11, >50 years: 18/32). Approximately 11.6% (*n* = 5) of PwTNs exhibited LDP in the QSART. No abnormal results were detected in the SSR test for PwTNs. Approximately 4.3% (*n* = 2) and 8.6% (*n* = 4) of PwTNs showed abnormal results in HRV and VR, respectively. Approximately 6.5% (*n* = 3) of patients showed abnormalities in both the QSART and other AFTs.

**Table 2:** Comparison of QSART results between tinnitus and control groups.

**Age group**	**Sex**	**Tinnitus**	**Control**	***p*-value**
**30**~**49**	**(M:F)**	***N** =* **11 (7:4)**	***N** =* **58 (28:30)**	
Arm	M	1.05 (0.17, 1.47)	0.40 (0.18, 1.00)	0.263
		F	0.40 (0.21, 1.72)	0.17 (0.12, 0.33)	0.064
Prox. Leg	M	1.56 (1.49, 1.78)	1.36 (0.91, 2.03)	0.732
		F	0.56 (0.26, 1.31)	0.63 (0.46, 0.98)	0.738
Dis. Leg	M	1.55 (1.01, 1.88)	1.16 (0.69, 1.51)	0.184
		F	0.13 (0.05, 0.90)	0.43 (0.30, 0.67)	0.162
Foot	M	0.80 (0.70, 0.99)	0.53 (0.22, 1.51)	0.017
		F	0.07 (0.01, 0.29)	0.14 (0.08, 0.28)	0.392
>**50**		***N** =* **32 (14:18)**	***N** =* **64 (27:37)**	
Arm	M	1.50 (0.80, 1.84)	1.00 (0.46, 1.85)	0.185
		F	0.50 (0.32, 1.38)	0.30 (0.18, 0.66)	0.013
Prox. Leg	M	1.54 (1.31, 1.82)	1.37 (0.97, 1.74)	0.541
		F	0.84 (0.50, 1.28)	0.58 (0.38, 0.79)	0.060
Dis. Leg	M	1.36 (1.00, 1.81)	1.39 (0.79, 1.87)	0.881
		F	0.58 (0.32, 0.84)	0.38 (0.23, 0.67)	0.116
Foot	M	1.05 (0.67, 1.92)	0.44 (0.20, 0.77)	< 0.001
		F	0.65 (0.33, 1.07)	0.13 (0.05, 0.26)	< 0.001

### 3.4 Comparison of QSART results between the age and sex-matched subgroups

**Figure 1:**
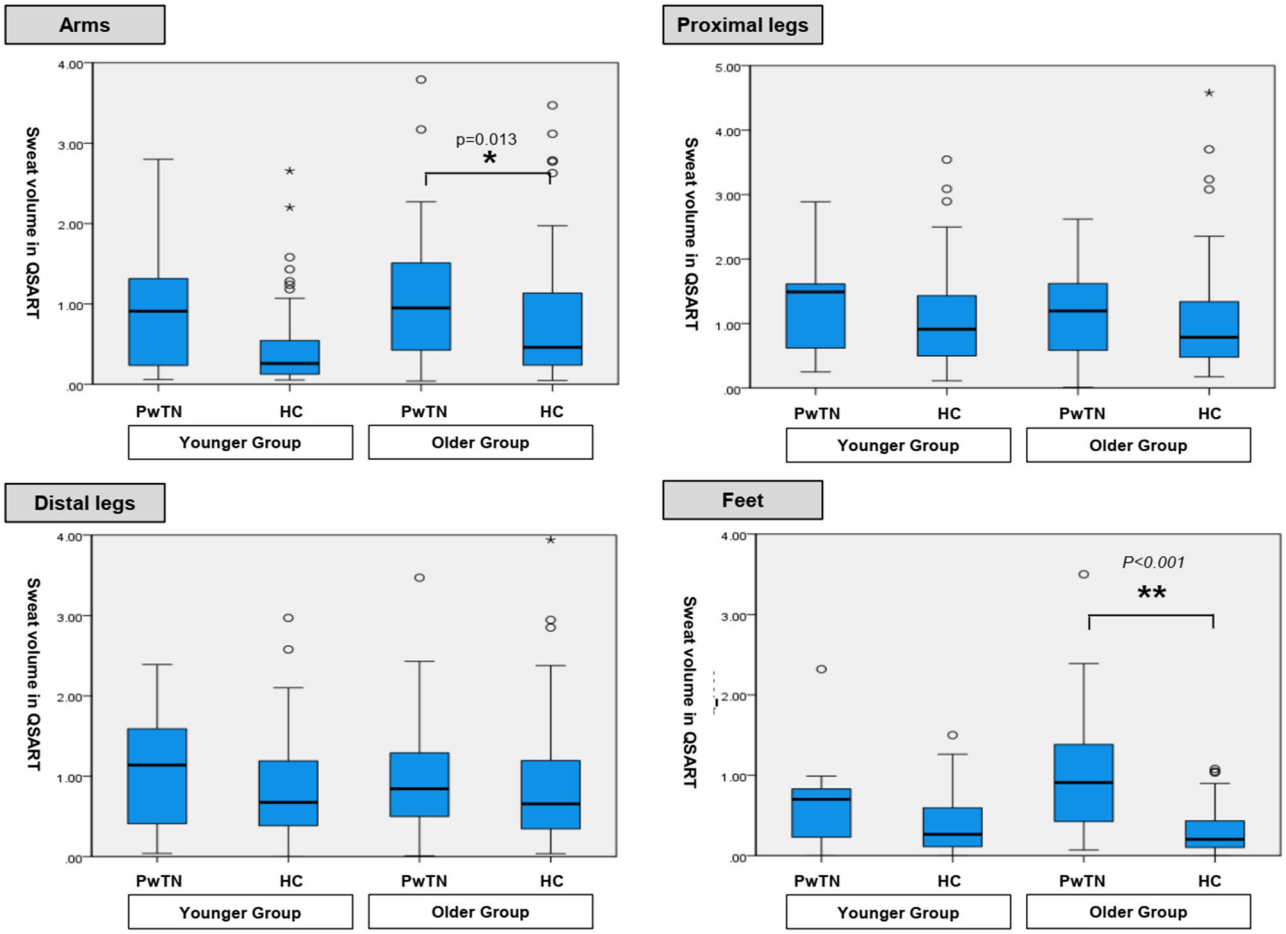
Comparison of sweat volumes in QSART between age-matched groups with tinnitus and healthy controls.

In the younger age group, sweat volumes in the feet in men with TN was significantly higher than that in healthy controls (*p* = 0.017). No statistically significant differences were observed at other sites. In the older age group, the sweat volume was significantly higher in the PwTN group than in the healthy control group in the feet in both sexes (*p* < 0.001), and in the arm in women (*p* = 0.013) (Table 2, Figure 1).

### 3.5 Association with sweat volumes in QSART and the results of audiometry

The number of SNHL was 79% (*n* = 34) in PwTN. The mean of AC threshold was 32.2 (Standard deviation, SD 19.5) on the right and 29.9 (SD 16.3) on the left. There was no association between the sweat volumes in QSART and the results of PTA.

## 4 Discussion

In this study, we performed the QSART to PwTN, comparing the results of the healthy controls. To our knowledge, this is the first study to check the function of the sympathetic domain and small-diameter nerve fibers using QSART and other AFTs to confirm dysautonomia in PwTNs. The QSART results revealed that sweat volumes in the PwTN were higher than those in the healthy control group, and this difference was more evident in the older age group. More than half of PwTNs showed abnormal QSART results. However, abnormal HRV and VR results were rarely observed. Also, we collected THI scores in PwTNs, and there were no relationships between THI scores and parameters in AFT.

Patients with TN frequently present at outpatient clinics of the Department of Neurology and Otorhinolaryngology. The underlying mechanism of TN remains unclear. In previous studies about TN, patients with DM are more likely to experience TN than healthy individuals of the same age. The incidence of TN increases with the duration of DM, possibly as a result of increased neurosensory impairment, such as diabetic neuropathy (24). In addition, few case reports on SFN and TN in patients with COVID-19 have been documented (6, 7, 12).

Increased sweat volume can occur under conditions of PN, SFN, and sympathetic dysfunction (25). It has also been reported that TN is frequently observed alongside autonomic dysfunction in older patients (26). In our study, none of the participants had DM; nonetheless, sweat volumes in PwTNs were higher than those in healthy controls. Thus, TN may be associated with axonal excitability similar to neuropathy or autonomic dysfunction. As the QSART is usually used to evaluate postganglionic sympathetic function, its association with dysautonomia cannot be ruled out (27).

In previous studies, dysautonomia in PwTNs was reportedly caused by ANS dysregulation, which is associated with chronic stress. From this perspective, decreased HRV and a relative predominance of sympathetic function may occur in PwTNs (15). Although the abnormalities in QSART results have not been studied in stress-related disorders, the effects of stress have been studied using HRV tests. HRV abnormalities have been observed in patients with fibromyalgia in response to stress (28). The association between stress and HRV abnormalities suggests abnormal sympathetic dominance (29). Although only 10% of PwTNs in our study directly reported experiencing stress, this could not guarantee low levels of stress in PwTN. Reflecting the scores of THI, in which half of PwTNs showed a low grade of disability for TN. It can be suggested that PwTNs are exposed to chronic stress by TN.

However, ~10% of PwTNs showed abnormal results in parasympathetic AFTs and abnormal QSART results were observed in more than half of PwTNs. It can be assumed that there is another mechanism apart from dysautonomia in the QSART abnormalities observed in PwTNs. Considering QSART evaluates the function of small fiber nerves, one of the mechanisms of TN may be the dysfunction of small fiber nerves related to the inner ear, such as the cochlear nerve.

SFN is defined as a dysfunction of small A delta and C fibers, suggesting distal axonal loss or neuronal degeneration rather than demyelinating process (16). Also, the degeneration of cochlear neurons or the loss of inner and outer hair cells was also reported as a possible mechanism for TN. SFN and TN may share these mechanisms such as neuronal degeneration or axonal loss (30, 31). Further larger studies will be needed in the future.

Our study has several limitations. First, the number of patients in the PwTN group was small. We enrolled consecutive PwTNs for 4 months. However, as the number of patients presenting to the clinic decreased during the lockdown period of the COVID-19 pandemic, the final number of patients decreased. Unlike PwTNs, since healthy controls were enrolled before the COVID-19 pandemic, it could have affected the results of this study by different enrolled times between PwTN and healthy controls. Second, we did not include patients aged < 30 years. TN is typically associated with age and is relatively uncommon in patients aged < 30 years. This limitation is caused by the characteristics of the conditions. Third, the QSART, SSR, HRV, and VR were conducted at different times in different places. Even though they followed the same protocols, it could have affected the results of AFTs. In addition, HRV and VR were calculated using a nerve conduction study system, which differed from the method used for the healthy control group. Therefore, HRV and VR could not be compared between PwTNs and healthy controls and abnormalities were judged, based on the reference values. More rigorous setup and statistical analysis, including correction for multiple comparisons, will be needed in further steps. At last, the audiometry was not performed for healthy controls. Although we enrolled the controls without any diseases, it is likely that the hearing ability was not matched between the two groups.

In conclusion, abnormal QSART results were frequently observed in PwTNs, and only a small number of patients exhibited abnormalities in HRV or VR. TN has been suggested that TN can occur alongside SFN in addition to autonomic dysfunction as a comorbidity. This is the first study to perform QSART with other parasympathetic AFTs in PwTNs. However, larger and more rigorously controlled studies are required to confirm this association in the future.

## Data availability statement

The raw data supporting the conclusions of this article will be made available by the authors, without undue reservation.

## Ethics statement

The studies involving humans were approved the Korea University Institution Research Board (K2023-1477-002). The studies were conducted in accordance with the local legislation and institutional requirements. Written informed consent for participation was not required from the participants or the participants' legal guardians/next of kin in accordance with the national legislation and institutional requirements.

## Author contributions

HLL: Formal analysis, Writing—original draft. HJL: Data curation, Writing—original draft. J-JS: Conceptualization, Writing—review & editing. CK: Funding acquisition, Supervision, Writing—review & editing.
